# Digital Cognitive Behavioral Therapy for Insomnia for Adolescents With Mental Health Problems: Feasibility Open Trial

**DOI:** 10.2196/14842

**Published:** 2020-03-03

**Authors:** Bethany Cliffe, Abigail Croker, Megan Denne, Jacqueline Smith, Paul Stallard

**Affiliations:** 1 Department of Health University of Bath Bath United Kingdom; 2 Child and Adolescent Mental Health Service Oxford Health NHS Foundation Trust Temple House Keynsham United Kingdom

**Keywords:** insomnia, internet-based intervention, cognitive therapy, mental health

## Abstract

**Background:**

Insomnia in adolescents is common, persistent, and associated with poor mental health including anxiety and depression. Insomnia in adolescents attending child mental health services is seldom directly treated, and the effects of digital cognitive behavioral therapy (CBT) for insomnia (CBTi) on the mental health of adolescents with significant mental health problems are unknown.

**Objective:**

This open study aimed to assess the feasibility of adding supported Web-based CBT for insomnia to the usual care of young people aged 14 to 17 years attending specialist child and adolescent mental health services (CAMHS).

**Methods:**

A total of 39 adolescents with insomnia aged 14 to 17 years attending specialist CAMHS were assessed and offered digital CBTi. The digital intervention was Sleepio, an evidence-based, self-directed, fully automated CBTi that has proven effective in multiple randomized controlled trials with adults. Self-report assessments of sleep (Sleep Condition Indicator [SCI], Insomnia Severity Scale, and Web- or app-based sleep diaries), anxiety (Revised Child Anxiety and Depression Scale [RCADS]), and depression (Mood and Feelings Questionnaire [MFQ]) were completed at baseline and post intervention. Postuse interviews assessed satisfaction with digital CBTi.

**Results:**

Average baseline sleep efficiency was very poor (53%), with participants spending an average of 9.6 hours in bed but only 5.1 hours asleep. All participants scored less than 17 on the SCI, with 92% (36/39) participants scoring 15 or greater on the Insomnia Severity Scale, suggesting clinical insomnia. Of the 39 participants, 36 (92%) scored 27 or greater on the MFQ for major depression and 20 (51%) had clinically elevated symptoms of anxiety. The majority of participants (38/49, 78%) were not having any treatment for their insomnia, with the remaining 25% (12/49) receiving medication. Sleepio was acceptable, with 77% (30/39) of the participants activating their account and 54% (21/39) completing the program. Satisfaction was high, with 84% (16/19) of the participants finding Sleepio helpful, 95% (18/19) indicating that they would recommend it to a friend, and 37% (7/19) expressing a definite preference for a digital intervention. Statistically significant pre-post improvements were found in weekly diaries of sleep efficiency (*P*=.005) and sleep quality (*P*=.001) and on measures of sleep (SCI: *P*=.001 and Insomnia Severity Index: *P*=.001), low mood (MFQ: *P*=.03), and anxiety (RCADS: *P*=.005).

**Conclusions:**

Our study has a number of methodological limitations, particularly the small sample size, absence of a comparison group and no follow-up assessment. Nonetheless, our findings are encouraging and suggest that digital CBTi for young people with mental health problems might offer an acceptable and an effective way to improve both sleep and mental health.

**International Registered Report Identifier (IRRID):**

RR2-10.2196/11324

## Introduction

### Adolescent Insomnia and Mental Health

Insomnia is a chronic dissatisfaction with sleep quantity or quality, despite an adequate opportunity to sleep [1]. It includes difficulties initiating, maintaining, or returning to sleep, with the sleep disturbance occurring at least three nights per week for 3 months and causing impairment in daytime functioning. It is the most prevalent adolescent sleep disorder, with community surveys identifying up to one-third of adolescents with significant symptoms of insomnia [2]. The mean age of onset of insomnia is 11 years, with symptoms being associated with significant mental health problems and tending to be persistent [3-5]. Associations have been found between insomnia and increased risk of nonsuicidal self-injury [6], poor school performance [7], anxiety, mood disorders, behavioral difficulties, substance use, eating disorders, and suicidality [2].

The mechanisms underlying these relationships are complex and potentially involve sequential, parallel, and interacting biological, psychological, and social systems [8]. Although there is a bidirectional relationship between sleep and mental health [5], there is evidence to suggest that sleep difficulties may precede the development of depression and anxiety [8-10]. Given the common comorbidity of insomnia across mental health diagnoses, interventions directly targeting sleep difficulties could have a positive effect on mental health outcomes [11].

### Cognitive Behavioral Therapy for Insomnia

Within an adult population, cognitive behavioral therapy for insomnia (CBTi) is an effective and recommended intervention for insomnia [12]. It is the first line of treatment offered for adults with insomnia and has been shown to be superior to pharmacological treatments in the long term [13]. CBTi involves a range of cognitive and behavioral techniques such as stimulus control, relaxation training, sleep restriction, sleep hygiene, and cognitive techniques to manage worries and intrusive thoughts. Although traditionally provided as a face-to-face intervention, CBTi is also effective when delivered digitally [14,15]. Randomized controlled trials with community cohorts of adults have shown that digital CBTi can improve both sleep and mental health, with reductions in symptoms of anxiety, depression, and psychosis being reported [16-18].

### Cognitive Behavioral Therapy for Insomnia for Children and Adolescents

The evidence base for children is more limited, although cognitive and behavioral interventions for insomnia for children in medical settings have shown promising results [19]. However, studies evaluating CBTi within mental health settings with adolescent clinical populations are extremely limited [20]. In a small pilot study, adolescents with a diagnosis of depression and insomnia were provided with 10 sessions of face-to-face CBTi (n=20) or sleep hygiene (n=21) in addition to CBT for depression [21]. Improvements in total time asleep and depression outcomes in the CBTi group were noted at 6 months. Similarly, in an open trial, adolescents (N=46) with depression and insomnia received a group CBTi intervention consisting of five 90-min sessions, which resulted in postintervention improvements in sleep and mood [22].

In terms of digital CBTi with adolescents, a recent systematic review found postintervention improvements in sleep efficiency, quality, total sleep time, and sleep onset latency [23]. However, the authors were only able to identify three studies that met their inclusion criteria, with one of these involving young people aged 19 to 34 years. In the largest study to date, a community study of Dutch adolescents (n=116) found that face-to-face and internet-delivered CBTi were both effective in improving sleep, with improvements being maintained at 1-year follow-up [24,25]. The authors also noted improvements in mental health, with reductions in affective and anxiety symptoms being mediated by improvements in sleep. No studies have yet evaluated the impact of digital CBTi on the sleep and mental health of adolescents with significant mental health problems attending specialist mental health services.

### Aims of the Study

The aim of this study was to evaluate the feasibility of adding supported, low-intensity, digital CBTi to the usual care of adolescents with significant mental health problems attending specialist child and adolescent mental health services (CAMHS). To determine feasibility, we collected data on participants’ perceived acceptability of Sleepio and assessed any impact on sleep (primary outcome) and mental health (secondary outcome).

## Methods

### Study Design

This was a pragmatic, pre-post, uncontrolled, mixed method, open feasibility study. A detailed methodology can be found in the study protocol [26].

### Participants

The participants were young people aged 14 to 17 years with mental health problems and comorbid symptoms of insomnia. Participants were recruited from child and adolescent mental health outpatient clinics under the Oxford Health National Health Services Foundation Trust across Bath and North East Somerset, Swindon, and Wiltshire.

### Exclusion Criteria

Young people were excluded if they presented with active suicidal ideation; they were diagnosed with psychosis; there were safeguarding concerns (ie, the young person had suffered abuse within the last 6 months or was the subject of a safeguarding investigation); and they had a significant developmental disorder (eg, autism), which prevented them from understanding the materials.

### Screening

Potential participants were screened by their clinician for sleep efficiency and motivation. Sleep efficiency was chosen as this is also the measure that is used within the digital CBTi Sleepio program to monitor progress and classify sleep. Young people reported the average amount of time they spent in bed and the amount of time spent asleep each night. Their sleep efficiency (time in bed divided by time asleep) was calculated. Adolescent sleep efficiency in a community sample (N=10,220) of adolescents aged 16 to 18 years was 85% to 88% [27]. A sleep efficiency of 85% or less was, therefore, required for participation.

The young person’s motivation to improve their sleep is an important factor that will affect their willingness to engage with sleep programs and to secure long-term sleep change [28,29]. Motivation was assessed by three items relating to problem severity (“At present, sleep is a big problem for me”), desire to change (“I want to change my sleep”), and self-efficacy (“I feel I can change my sleep”). Each item was rated on a 10-point Likert scale ranging from 0 (strongly disagree) to 10 (strongly agree), with a score of 5 or more on each item being required for participation.

### Enrollment and Consent

A researcher met with interested individuals (and their parents if aged <16 years) to explain the project. Those aged 14 to 15 years provided signed assent and their parents signed consent. Those aged 16 to 17 years provided signed consent.

### Ethics

The study was approved by the South West-Central Bristol Research Ethics Committee (17/SW/0178). Any face-to-face intervention or medication participants were receiving through CAMHS was continued, and their CAMHS clinician continued to take responsibility for the young person’s care.

### Intervention

Sleepio is an established, fully automated, Web-based, self-administered sleep intervention that has been evaluated with adults [18,30,31]. As this study aimed to assess the acceptability of Sleepio for young people, no developmental adaptations were made to the existing program. The intervention was free to study participants and consisted of 6 sessions, each lasting approximately 20 min, which were released each week. The sessions were accessed via a Web browser on a tablet, desktop, or mobile phone. The program is highly interactive, and content is presented via an animated cartoon therapist (*The Prof*). Participants completed daily Web or app-based sleep diaries throughout the program, with an algorithm personalizing the content of the program to the individual’s needs. Sleepio is based on CBTi and incorporates cognitive (paradoxical intention, cognitive restructuring, mindfulness, positive imagery, and putting the day to rest), behavioral (sleep restriction, stimulus control, and relaxation), and educational (sleep hygiene and the process of sleep) components. Participants took an average of 8.5 weeks to complete the program.

Engagement with Web-based self-help mental health programs is variable and is typically lower than that with guided interventions [11]. As Sleepio has not previously been used with this age group, we augmented the programs with brief (up to 15 min), weekly support telephone calls from a trained Sleepio assistant. The support calls were designed to maintain motivation and engagement and followed a similar process to that used by Luik et al [31]. They focused solely on how the material presented in each Sleepio session could be applied to the young person’s circumstances and did not address the wider mental health of the young person.

### Acceptability of Sleepio

A semistructured interview was undertaken with young people to gather detailed feedback on their experience of Sleepio and their perceived acceptability of it. Qualitative questions assessed how, where, and how often Sleepio was accessed; which techniques and sessions were most useful; experience of using a Web-based program and telephone support; and how the program could be improved. The frequencies of reported themes were summarized, and key quotes were extracted to contextualize the results. In addition, young people rated on a 4-point Likert scale the ease of use, helpfulness, preference over face-to-face meetings, whether sessions were understandable, and if they would recommend Sleepio to a friend. Perceived changes in sleep, mental health, and overall satisfaction were rated on a 10-point Likert scale.

### Outcome Measures

Standardized questionnaires were completed at baseline and on completion of the program.

#### Sleep

Sleep was assessed using the Insomnia Severity Index (ISI) [32] and the Sleep Condition Indicator (SCI) [33]. The ISI is a 7-item self-report measure assessing symptoms of insomnia over a 2-week period on a 5-point scale. The ISI assesses sleep onset, sleep maintenance, early morning awakening problems; sleep dissatisfaction; interference of sleep difficulties with daytime functioning; whether sleep problems are noticed by others; and distress caused by sleep difficulties. The ISI has excellent internal consistency (Cronbach alpha=.91), with a cutoff of 15 or greater identifying 88% of those diagnosed with insomnia [32].

The SCI is an 8-item self-report measure assessing sleep and its impact on daytime functioning over the previous month on a 4-point scale. The SCI assesses sleep continuity (falling and remaining asleep), satisfaction with sleep (quality and troubled by sleeping), severity (nights per week and problem duration), and consequences of poor sleep (impact on personal functioning and performance). The SCI is internally consistent (Cronbach alpha=.86), with a clinical cutoff of less than 17 correctly identifying 89% of those with probable Diagnostic and Statistical Manual of Mental Disorders (DSM), Fifth Edition, insomnia disorder [30,33].

#### Anxiety

Symptoms of anxiety were assessed using the Revised Child Anxiety and Depression Scale (RCADS) [34]. The RCADS is a 47-item questionnaire assessing DSM, Fourth Edition, criteria for social phobia, separation anxiety, obsessive compulsive disorder, panic disorder, generalized anxiety disorder, and major depressive disorder. Each item is rated on a 4-point Likert scale of frequency ranging from never (0) to always (3), and items are then summed to produce subscale and total anxiety scores. There are age- and gender-related norms for identifying clinically significant scores (*t* scores ≥65).

#### Depression

Symptoms of depression were assessed using the Mood and Feelings Questionnaire (MFQ) [35]. The MFQ consists of 33 items, each rated as either true (scores 2), sometimes true (scores 1), or not true (scores 0). The MFQ has high criterion validity and correlates well with other measures of depression. A total score of 27 or greater is associated with major depression, 17 to 26 with mild depression, and 16 or less with no mood disorder.

### Statistical Analysis

Descriptive statistics summarize the cohort, with group differences being examined by Student's *t* test.

## Results

### Participant Flow

A total of 50 young people were screened, with one being unable to participate because of becoming homeless. The remaining 49 participants were enrolled and contacted to arrange a baseline assessment. One young person did not respond; 3 participants had other commitments they needed to prioritize; 2 participants no longer wanted to be involved; and the mental health of 2 participants had deteriorated with the sleep of 2 participants improving. Baseline assessments were completed with the remaining 39 participants. Of the 39 participants, 30 (77%) assessed at baseline completed at least one Sleepio session, 22 (56%) completed at least two sessions, 21 (54%) completed at least three sessions, 16 (41%) completed at least five sessions, and 13 (33%) completed at least six sessions. The average number of Sleepio sessions completed was 3.93 (SD 2.16). Postuse assessments were undertaken with 19 of 30 (63%) participants who engaged with the program, and interviews about the experience and acceptability of the program were conducted with 12 of 30 (40%) participants.

### Participant Characteristics

The 49 participants enrolled were predominantly female (37/49, 76%), with an average age of 15.6 (SD 1.19) years. The group had poor sleep efficiency (52%, SD 13.59) and spent an average of 9.6 (SD 1.8) hours in bed each night, of which they were asleep for 5.1 (SD 1.6) hours. Self-report ratings out of 10 indicated that sleep was a big problem (mean 8.2, SD 1.6), which they wanted to change (mean 9.1, SD 1.2) and indicated that they felt they were able to change (mean 6.3, SD 1.2).

The primary diagnoses of those enrolled were emotional disorders of anxiety or depression (37/49, 76%), with almost half (22/49, 45%) of the participants reporting a history of self-harm. Pharmacological interventions were prescribed for 51% (25/49) young people, with 37% (18/49) taking selective serotonin reuptake inhibitors (SSRIs; fluoxetine, n=11; sertraline, n=5; and citalopram, n=2) and 25% (12/49) receiving a pharmacological intervention for their sleep (melatonin, n=3; zopiclone, n=1; and circadian, n=8). None of the participants had received a psychological sleep intervention.

There were no differences between those who went on to complete baseline assessments (n=39) and those who did not (n=10) in gender (Levene test indicated unequal variances, so degrees of freedom were adjusted [*t*_19.8_=1.47; *P=*.16]), age (*t*_47_=−0.04; *P=*.97), sleep being a big problem (*t*_47_=0.22; *P=*.83), wanting to change sleep (*t*_47_=1.47; *P=*.15), or feeling able to change sleep (*t*_47_=1.68; *P=*.10). There was one difference in sleep (*t*_47_=3.35; *P=*.002), with those who completed baseline assessments spending almost 2 hours more in bed (mean 9.95 hours, SD 1.66) than those who did not (mean 8.05 hours, SD 1.30).

Demographic and assessment data for the 39 participants who completed baseline assessments are summarized in Table 1.

All participants scored less than 17 on the SCI, with 92% (36/39) participants scoring 15 or greater on the Insomnia Severity Scale, suggesting clinical insomnia. Sleep difficulties were regular and chronic, with 95% (37/39) participants reporting problems sleeping for 4 or more nights each week and 69% (27/39) participants reporting difficulties for more than 1 year. Poor sleep severely, or very severely, interfered with everyday functioning (35/39, 90%); caused problems with concentration, productivity, or ability to stay awake (34/39, 87%); and affected mood, energy, or relationships (31/39, 80%). Satisfaction with sleep was poor, with 95% (37/39) participants being severely, or very severely, dissatisfied with their current sleep pattern.

**Table 1:** Participant demographics and baseline sleep, motivation, anxiety, and depression (N=39).

Characteristic		Values
Age (years), mean (SD)		15.6 (1.21)
**Gender, n (%)**		
	Female	28 (72)
	Male	11 (28)
Average number of hours in bed each night, mean (SD)		9.95 (1.66)
Average number of hours asleep per night, mean (SD)		5.15 (1.23)
Average percentage of sleep efficiency=hours asleep/hours in bed, mean (SD)		52.86 (13.56)
Sleep is a big problem—rating out of 10, mean (SD)		8.18 (1.49)
I want to change my sleep—rating out of 10, mean (SD)		8.97 (1.29)
I feel I can change my sleep—rating out of 10, mean (SD)		6.13 (1.08)
**Primary diagnosis, n (%)**		
	Anxiety disorder	11 (28)
	Depressive disorder	8 (21)
	Mixed anxiety and depressive disorder	13 (33)
	Eating disorder	3 (8)
	Posttraumatic stress disorder	2 (5)
	Autism spectrum disorder	2 (5)
**History of self-harm, n (%)**		
	Yes	18 (46)
	No	21 (54)
**Currently prescribed medication, n (%)**		
	Yes	22 (56)
	No	17 (44)
**Currently prescribed sleep medication, n (%)**		
	Yes	10 (26)
	No	29 (74)
Sleep Condition Indicator, mean (SD)		6.56 (3.04)
Insomnia Severity Index, mean (SD)		19.64 (3.54)
**Revised Anxiety and Depression Scale, mean (SD)**		
	Obsessive compulsive disorder	7.05 (3.76)
	Separation anxiety	8.69 (4.78)
	Social anxiety	18.82 (6.23)
	Generalized anxiety	9.95 (4.21)
	Panic disorder	12.79 (6.46)
	Depression	20.21 (4.62)
	Anxiety total	57.03 (20.26)
	Total Revised Child Anxiety and Depression Scale	77.23 (23.52)
Mood and Feeling Questionnaire, mean (SD)		42.05 (11.70)

In terms of mental health, the group presented with significant symptoms of depression and anxiety. On the MFQ, 92% (36/39) participants scored 27 or greater, suggesting major depression. Of the 39 participants, 16 (41%) endorsed the statement “I thought that life wasn’t worth living,” 15 (39%) endorsed the statement “I thought about death or dying,” and 13 (33%) endorsed the statement “I thought about killing myself.” Similarly, there were significant symptoms of anxiety. Using age- and gender-adjusted norms, 51% (20/39) participants scored within the clinical range (*t* score≥70) for the total RCADS, with a further 23% (9/39) participants falling within the borderline range (*t* score=65).

A comparison of baseline data was undertaken for those who completed follow-up assessments (n=18) and those who did not (n=21). There were no differences in age (*t*_37_=−0.34; *P=*.73); gender (*t*_37_=−0.25; *P=*.81); perception of sleep as a problem (*t*_47_=0.52; *P=*.61); desire to change sleep (*t*_37_=1.94; *P=*.06); ability to change sleep (*t*_37_=−0.46; *P=*.65); or baseline scores on standardized measures of sleep (SCI: *t*_37_=0.60; *P=*.55 and ISI: *t*_37_=−0.16; *P=*.87), depression (MFQ: *t*_37_=0.79; *P=*.44), or anxiety (RCADS: *t*_37_=−0.33; *P=*.74).

### Acceptability of Sleepio

Semistructured interviews were undertaken with 12 young people. Most young people accessed Sleepio at least once per week (10/12, 83%), via a laptop (10/12, 83%), in their room (11/12, 92%), and on their own (11/12, 92%). Young people were positive about working with the Web-based program (10/12, 83%) and identified a number of benefits. Working on the Web was seen as less stressful (5/12, 42%): “It was less stressful, like I could do it when I wanted” and “made it a little less intimidating cos you’re not talking to someone.” Other benefits were convenience (4/12, 33%): “you could go through it in your own time, there wasn’t any pressure.” Three young people highlighted that they found face-to-face meetings difficult and so welcomed the Web-based intervention: “I am rubbish at speaking to people, it’s exhausting, so I like this.” Of the 2 participants who expressed a preference for face-to-face meetings, both highlighted that they missed the opportunity to ask questions and to seek clarification of the things they did not understand: “face to face is probably better, you can ask questions if you don’t understand.”

The weekly support telephone calls were used by 16 out of the 30 young people who, on average, received a total of 38 min during the course of the program. Of the 14 participants who did not take up the telephone support, 7 dropped out after session 1 compared with 1 of the 16 participants of those who received support. Of those interviewed, 11 out of the 12 young people used the telephone support. Although all the participants found the calls helpful, 3 did not think they were necessary: “I think it would’ve been fine without the phone calls though, because I never had any questions or anything.” The reported benefits of the calls included being able to recap on sessions (9/12, 75%): “I think it was good to just talk over what you’d done.” Five participants welcomed the opportunity to ask questions: “It was good if I had any questions,” with 2 young people finding the calls motivating: “It definitely kept me more motivated to do it cos it’s an actual person.”

In terms of the specific techniques that were helpful, sleep restriction (reducing the amount of time spent in bed to consolidate sleep) was identified most often (6/12, 50%). Although helpful, it was also seen as challenging: “It was hell on earth, but it was useful, it did help definitely.” However, another 3 participants identified sleep restriction less positively, finding it hard to implement: “If I was really tired, I would go to bed before Sleepio said I was supposed to.”

The 15-min rule (ie, if awake for 15 min, get out of bed) was positively endorsed by 5 out of the 12 young people. One young person commented: “I tend to work myself up quite a lot so having the 15 minutes rule if I haven’t slept to just get out of that working myself up thing.” Another young person did not find this helpful and reported the opposite effect: “I kind of gave up with it after a week cos it just stresses you.”

Relaxation was identified as helpful by 4 out of the 12 young people: “Imagery and progressive relaxation were good for taking my mind off of other things.” Another young person did not find progressive relaxation helpful (“It wasn’t that things weren’t useful, they just didn’t work for me”), and one person did not like mindfulness (“mindfulness I didn’t like”). Finally, 2 young people identified thought blocking and 1 identified sleep hygiene as helpful.

In terms of improvements, 3 young people commented about issues with connectivity (“Tiny thing was that it got a bit glitchy at some points when you were doing the sessions”), although all participants noted that this may have been because of their internet connection (“I think it might just have been my rubbish Wi-Fi”). One participant requested sessions to be released earlier rather than waiting a week; another participant requested short-time interval in the sleep diary (5 min blocks instead of 15 min); one participant requested wanting to print out material; and one participant requested sleep diary reminders. Only 1 young person commented on improving the program content and how thought blocking and mindfulness were unhelpful.

### Satisfaction and Helpfulness

A total of 19 young people completed Likert ratings of satisfaction and helpfulness. Overall satisfaction, assessed on a 1 to 10 scale, was good (mean 6.89, SD 1.97). Participants found Sleepio easy to use (14/19, 74%) and understand (16/19, 84%), with just under half (9/19, 47%) of the participants definitely finding it helpful and a further 37% (7/19) participants finding it partially helpful. In terms of endorsing Sleepio, only 5% (1/19) participants would not recommend Sleepio to a friend, and 68% (13/19) participants would definitely recommend it.

On a 10-point scale, the mean rating of sleep improvement was 5.58 (SD 2.22), but it was lower, 3.37 (SD 2.54), for perceived improvements in mental health. In terms of delivery, 37% (7/19) participants expressed a definite preference for the Web-based program, with 26% (5/19) participants stating a definite preference for a face-to-face intervention.

### Postuse Outcome Data

Pre-post data are summarized in Table 2.

**Table 2:** Pre- and postdata (N=19).

Psychological measure		Baseline, mean (SD)	Postuse, mean (SD)	95% CI	*P* value
**RCADS^a^**					
	Obsessive compulsive disorder	6.58 (3.44)	5.42 (4.19)	−0.25 to 2.57	.10
	Separation anxiety	8.53 (4.39)	6.05 (4.33)	1.22 to 3.73	*.001* ^b^
	Social anxiety	20.37 (5.64)	18.00 (5.74)	−0.15 to 4.89	.06
	Generalized anxiety	10.68 (4.04)	9.74 (4.53)	−0.55 to 2.44	.20
	Panic disorder	13.11 (5.50)	11.16 (6.27)	0.11 to 3.79	*.04* ^b^
	Depression	19.26 (4.81)	15.32 (6.05)	1.45 to 6.45	*.004* ^b^
	Anxiety total	59.26 (19.01)	50.37 (20.82)	2.59 to 15.20	*.008* ^b^
	Total RCADS	78.53 (23.06)	65.68 (26.17)	4.35 to 21.33	*.005* ^b^
Mood and Feeling Questionnaire		40.53 (13.13)	33.79 (16.34)	0.94 to 12.54	*.03* ^b^
Sleep Condition Indicator		6.26 (2.71)	15.11 (7.89)	−12.26 to −5.43	*<.001* ^b^
Insomnia Severity Index		19.74 (3.25)	12.47 (6.05)	4.87 to 9.66	*<.001* ^b^

^a^RCADS: Revised Child Anxiety and Depression Scale.

^b^Value reached significance.

#### Sleep

Paired *t* tests identified statistically significant postintervention changes on all measures. Sleep improved as assessed by the SCI (*t*_18_=−5.44; *P*<.001) and the ISI (*t*_18_=6.36; *P*<.001). Using the recommended cutoffs for identifying significant symptoms of insomnia, there was a significant change in both measures. On the SCI, there was a postintervention reduction from 19 to 10 in those scoring less than 17, and on the ISI, there was a reduction from 19 to 6 in those scoring 15 or greater. Finally, a comparison between the first and the last sleep diaries completed each week identified improvements in sleep quality (*t*_23_=−5.81; *P*<.001) and efficiency (*t*_24_=3.11; *P=*.005), with 69% of total time in bed spent asleep compared with 54% at baseline.

#### Mental Health

In terms of mental health, there were postintervention reductions in symptoms of depression (MFQ: *t*_18_=2.44; *P=*.03), with a slight postintervention reduction in the number scoring 27 or greater from 16 to 14. There were reductions in anxiety (total RCADS: *t*_18_=3.18; *P=*.005), which were particularly marked on the subscales assessing separation anxiety (*t*_18_=4.13*; P<.*001), panic (*t*_18_=2.22; *P=*.04), and depression (*t*_18_*=*3.32; *P=*.004). The number within the clinical range on the RCADS (*t* score≥70) reduced from 10 to 7, with a reduction from 15 to 7 of those scoring within either the borderline or clinical range (*t* score ≥65).

## Discussion

### Principal Findings

The adolescents who participated in this open feasibility study were attending specialist CAMHS, and all the adolescents presented with clinical levels of mental health problems. The group presented primarily with affective disorders, self-harm was common, and almost half of the participants were prescribed SSRIs. Insomnia was chronic, severe, and disabling; one-fourth of the participants were receiving a pharmacological intervention for their insomnia, and none of the participants had been offered a psychological intervention. Despite these high levels of psychopathology, our results are encouraging and demonstrate that brief, supported, digital CBTi was acceptable and was associated with reductions in symptoms of insomnia, increased sleep quality and efficiency, and fewer symptoms of anxiety and depression.

It proved feasible to recruit young people with comorbid insomnia from child mental health services. Program adherence was good, with 54% (21/39) of those who started digital CBTi completing more than half of the program sessions. However, this is significantly lower than the rates reported by others assessing digital CBTi with adolescents [24,25]. At baseline, the amount of time spent in bed was the only difference between those who activated their Sleepio account and those who did not. Although a formal comparison of Sleepio completers and noncompleters was not possible because of small numbers, we did observe some trends. Noncompleters were more likely to have a history of self-harm (9/9, 100%), a diagnosis of depression or mixed depression/anxiety (6/9, 67%), and be prescribed medication (5/9, 56%) than completers (self-harm, 7/21, 33%; depression/mixed depression, 9/21, 43%; and medication, 9/21, 43%). This needs to be explored in further studies but may indicate that supported digital CBTi may be more appropriate for adolescents with less complex mental health presentations.

Satisfaction with digital CBTi was high, with 95% (18/19) of the participants recommending digital CBTi to a friend. Of the 19 participants, 7 (37%) expressed a definite preference for the digital program, noting advantages of convenience, less stress, and an alternative nonverbal intervention. The advantage of a nonverbal delivery model was particularly highlighted during interviews by those who found face-to-face interventions difficult. However, 26% (5/19) of participants stated a definite preference for a face-to-face intervention as opposed to a digital intervention. Interviews with 2 young people who expressed this preference highlighted how they missed the interactive opportunity to ask questions. Further research is required to explore treatment preferences, and although digital sleep interventions may not be the first choice for all adolescents, our results suggest that some will have a clear preference for digital delivery.

In terms of program content, 9 of the 12 participants interviewed specifically commented about sleep restriction. Sleep restriction is a core component of CBTi, although there is considerable variation in the way the sleep window is calculated [36]. In our study, we did not make any developmental modifications and used the existing algorithm of the adult Sleepio program. Most (6/9, 67%) of the participants identified sleep restriction as helpful although challenging, with the remaining 3 of 9 participants reporting that they were unable to fully implement these techniques. We do not know whether this would have been easier to implement if sleep restriction was more specifically adapted for this younger age group. Although this should be explored in future studies, our preliminary findings suggest that the unmodified program did result in significant improvements in sleep efficiency and quality.

The digital intervention was low intensity, delivered in just over 2 hours, alongside the young person’s usual face-to-face mental health intervention. Telephone support from a trained graduate psychologist required an average of 38 min per participant and was viewed positively by most young people. In particular, they welcomed the usefulness of recapping on the session content and how they could apply the ideas to their situation. The motivational aspects of the support calls were identified by 2 young people, with others being unsure whether the support calls were necessary. However, the calls may have helped with initial engagement. Half of those (7/14) who did not take up the support call dropped out after session 1 compared with only 6% (1/16) of those who received the support. With such a small sample, it is unclear whether those who used telephone support were more motivated than those who did not. This suggests the need for a flexible approach where different levels of support may be required to engage with and complete digital interventions.

Our results are consistent with those undertaken with adults where Web-based CBTi has improved both sleep and mental health [16-18,30,37-40]. These findings are also consistent with community studies where Web-based CBTi for adolescents with a primary diagnosis of insomnia showed improvements in mental health outcomes including affective and anxiety symptoms [24,25]. Studies evaluating the use of CBTi with clinical groups are extremely limited, although findings from pilot studies have shown that face-to-face CBTi resulted in improvements in both sleep and mental health [21,22]. However, to our knowledge, this is the first study evaluating digital CBTi with adolescents with mental health problems attending specialist child mental health services. Despite the high levels of mental health psychopathology, postintervention assessments demonstrated that low-intensity digital CBTi was associated with improvements in sleep, anxiety, and depression. If substantiated in a robust, comparative, randomized controlled trial, digital CBTi may offer an accessible, low-cost, less stigmatizing way of providing mental health interventions for adolescents.

### Limitations

Although encouraging, our study suffers from a number of limitations that need to be acknowledged. First, our study was a small open feasibility study with no comparison group. Although our results are consistent with others, we do not know whether the improvements we report are because of Sleepio or the face-to-face CAMHS intervention the young person received. Second, digital CBTi involved telephone support, and it is possible that this contact may have confounded the results we report. Although feedback from young people identified that these calls remained focused on the CBTi session content, it is possible that this may have had a secondary effect on the outcomes we report. Third, our group was self-selected and highly motivated and may not, therefore, be representative of those attending child mental health services. Although a limitation, this nonetheless suggests that there is a group of motivated young people with chronic insomnia attending child mental health services who might benefit from the addition of digital CBTi. Fourth, we relied on self-reports and did not have any objective measures of sleep (eg, actigraphy) or undertake any follow-up assessments. Self-reports may suffer from recall bias and tend to overestimate total sleep compared with actigraphy [41]. Finally, we added digital CBTi to the usual care of young people already attending CAMHS, and we cannot generalize these findings outside of this setting.

### Conclusions

These results suggest that the unmodified Sleepio digital CBTi intervention is feasible to deliver to young people with significant mental health problems attending specialist mental health services. Digital CBTi was acceptable, with a number of young people expressing a preference for non–face-to-face interventions. Pre-post improvements in sleep, anxiety, and depression are encouraging and support the undertaking of a robust, suitably powered, randomized controlled trial to compare the effectiveness and cost-effectiveness of digital CBTi on the sleep and mental health of young people with identified mental health problems. This could be a 2-arm (supported digital CBTi vs usual CAMHS care) or a 3-arm (supported digital CBTi vs self-guided CBTi vs usual care) trial. This low-intensity intervention has the potential to be cost-effective and could be incorporated into a stepped care pathway where a brief digital CBTi could be provided before pharmacological interventions.

## Abbreviations

CAMHS: Child and Adolescent Mental Health Services
CBT: cognitive behavioral therapy
CBTi: cognitive behavioral therapy for insomnia
DSM: Diagnostic and Statistical Manual of Mental Disorders
ISI: Insomnia Severity Index
MFQ: Mood and Feelings Questionnaire
RCADS: Revised Child Anxiety and Depression Scale
SCI: Sleep Condition Indicator
SSRI: selective serotonin reuptake inhibitor
